# The stability and activity of human neuroserpin are modulated by a salt bridge that stabilises the reactive centre loop

**DOI:** 10.1038/srep13666

**Published:** 2015-09-02

**Authors:** Rosina Noto, Loredana Randazzo, Samuele Raccosta, Sonia Caccia, Claudia Moriconi, Elena Miranda, Vincenzo Martorana, Mauro Manno

**Affiliations:** 1 National Research Council of Italy, Institute of Biophysics, Palermo, Italy; 2 Department of Medical Biotechnologies and Translational Medicine, University of Milan, Milan, Italy; 3 Department of Biology and Biotechnologies “Charles Darwin” and Pasteur Institute - Cenci Bolognetti Foundation, Sapienza University of Rome, Rome, Italy

## Abstract

Neuroserpin (NS) is an inhibitory protein belonging to the serpin family and involved in several pathologies, including the dementia Familial Encephalopathy with Neuroserpin Inclusion Bodies (FENIB), a genetic neurodegenerative disease caused by accumulation of NS polymers. Our Molecular Dynamics simulations revealed the formation of a persistent salt bridge between Glu289 on strand s2C and Arg362 on the Reactive Centre Loop (RCL), a region important for the inhibitory activity of NS. Here, we validated this structural feature by simulating the Glu289Ala mutant, where the salt bridge is not present. Further, MD predictions were tested *in vitro* by purifying recombinant Glu289Ala NS from *E. coli*. The thermal and chemical stability along with the polymerisation propensity of both Wild Type and Glu289Ala NS were characterised by circular dichroism, emission spectroscopy and non-denaturant gel electrophoresis, respectively. The activity of both variants against the main target protease, tissue-type plasminogen activator (tPA), was assessed by SDS-PAGE and chromogenic kinetic assay. Our results showed that deletion of the salt bridge leads to a moderate but clear reduction of the overall protein stability and activity.

Human neuroserpin (NS) is a member of the serpin family (SERine Protease INhibitor)^1^. It is mainly secreted from neurons and, as an inhibitor of tissue-type plasminogen activator (tPA)^2^, it is involved in many physiological processes, such as synaptic plasticity, memory and the permeability of the neurovascular compartment^3^,^4^, as well as in neurological disorders^5^. NS is also directly involved in a severe genetic disease, the dementia Familial Encephalophaty with Neuroserpin Inclusion Bodies (FENIB), due to the aberrant polymerisation of NS within the endoplasmic reticulum^6^.

Beyond its relevance in FENIB, NS is an excellent model for studying serpins and the common pathways that may lead to serpinopathies, a class of serpin-related diseases^7^,^8^. Indeed, NS has a high structural analogy with the archetypal serpin *α*_1_-antitrypsin^9^,^10^; the polymerisation propensity of mutated NS variants is directly related to the severity of disease with a remarkable genotype-phenotype correlation^11^,^12^,^13^; moreover, NS polymerisation can be induced by thermal stress, even in the wild type (WT) form^14^,^15^,^16^,^17^,^18^,^19^.

After the determination of the crystal structure of the native and cleaved conformers of NS^9^,^10^, other studies have revealed the main structural features of monomeric and polymeric NS^20^,^21^,^22^,^23^. In a recent work^23^, we performed Molecular Dynamics (MD) simulations of different NS isoforms, finding that the conformational stability and flexibility of NS arise from a characteristic spatial distribution of intramolecular salt bridges and hydrogen bonds. The MD trajectory for native NS revealed the presence of a persistent salt bridge between the glutamic acid Glu289 on strand s2C in the main protein core and the arginine Arg362 located in the reactive centre loop (RCL), close to the cleavage site (P1-P1’). The RCL is a key structural element of all inhibitory serpins. It acts as a bait for the target protease, which cleaves the RCL forming an acyl-enzyme complex and triggering the insertion of the RCL as a new strand of the main central *β*-sheet. This mousetrap-like mechanism causes the translocation of the protease to the opposite side of the serpin and its disruption^24^.

The salt bridge between Glu289 and Arg362 was not observed in the available crystal structures of native NS, due to the intrinsic flexibility of the RCL. On the other hand, analogous motifs may be found in other serpins. In *α*_1_-antitrypsin, a salt bridge links Glu223 on strand s3C to Arg354 on the RCL, near to the clevage site (P1-P1’) at residues Met358-Ser359^25^.

Here, we performed the *in silico* mutation of Glu289 into Alanine, E289A, and back to glutamic acid, in order to validate the existence of this salt bridge and its statistical significance. Also, we performed the *in vitro* mutation E289A by site directed mutagenesis of WT NS. We have investigated the stability, conformation, function and dynamics of WT and E289A NS both *in silico* and *in vitro*. The deletion of the salt bridge 289–362 did not damage the fold and inhibitory capability of NS, however it affected its overall stability and dynamics, with a slight reduction of its inhibitory activity and its resistance against polymerisation.

## Results

### *In silico* mutagenesis of neuroserpin

**Figure 1:**
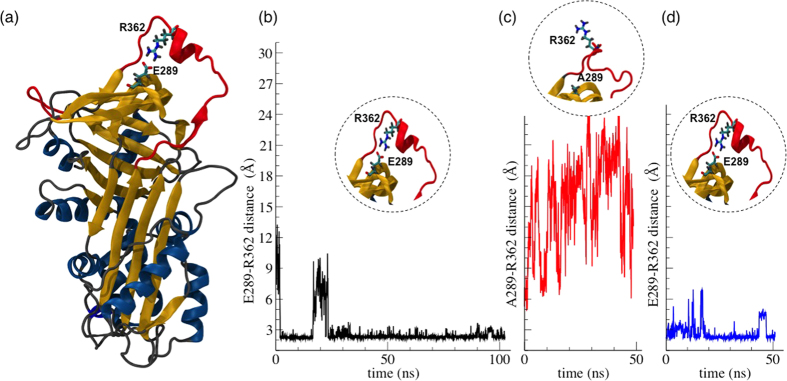
The alchemic mutation of E289A NS in MD simulations. (**a**) Cartoon representation of native NS; the lateral chains of residues E289 and R362 are shown in bond representation. (**b**–**d**) Time evolution of the distance between the selected residues highlighted in the circular insets: (**b**) E289-R362 (black line) in WT NS; (**c**) A289-R362 (red line) in E289A NS; (**d**) E289-R362 (blue line) in WT NS after the back mutation A289E;

In our previous work^23^, we reported a long MD simulation of native NS, which showed the formation of a persistent salt bridge between the arginine Arg362 on the RCL and the glutamic acid Glu289 on strand s2C in the main protein core (Fig. 1a). Figure 1b shows that the distance between the acid or basic groups of of the two residues is below 3 Å for most of the simulation run. Such a structural detail remained undetected in previous studies of NS.

### Validation of a structural feature revealed by MD by alchemical perturbation

Since this salt bridge was not evident in the available crystal structures^9^,^10^, we challenged its actual existence and performed an alchemical mutation of Glu289 to Alanine. This *in silico* mutation removes the salt bridge and alters the overall protein dynamics (Fig. 1c). After an adequately long simulation run, the inverse mutation A289E was performed by the same method. As shown in the distance trajectory of Fig. 1d, the salt bridge was soon restored (with high stability after 18 ns). The two-step mutation with elimination and restoration of the charged side chain of residue E289 was used to rule out any accidental dependence upon the initial coordinates. The stability of the bridge during the rest of the simulation (with some fluctuation observable after 43 ns), confirmed its robust persistence in the protein structure and allowed us to validate its statistical significance.

### Effects of the E289A mutation on the structure of neuroserpin

**Figure 2:**
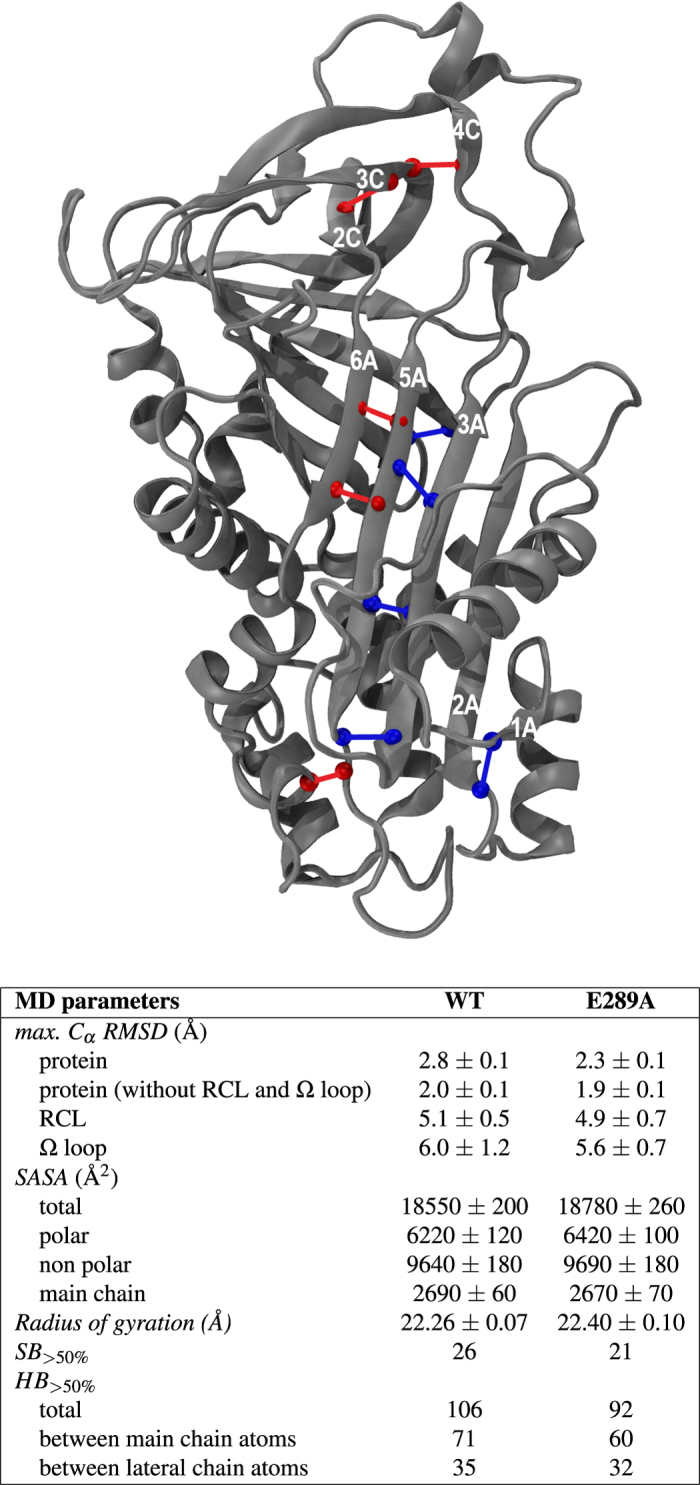
MD structural features. *Table:* MD parameters for WT and E289A NS: *C*_*α*_ Root Mean Square Displacement (RMSD) at the maximum stationary value, Solvent Accessible Surface Area (SASA), Radius of gyration (these quantities have been time-averaged over the last 20 ns simulation), Salt Bridges and Hydrogen Bonds with more than 50% time occupancy (*SB*_>50%_, *HB*_>50%_). *Picture:* Cartoon representation of native NS showing the main-chain hydrogen bonds (HB) uniquely present in one of the two NS with more than 50% occupancy. WT NS (blue ball and sticks): Arg383-Glu250 (s3B-s4B), Phe341-Val184 (s5A-s3A), Asn116-Val139 (2A-s1A), Asn182-Ile337 (s3A-s5A), Ser334-Leu178 (s5A-s3A); E289A NS (red ball and sticks): Ser340-Gln299 (s5A-s6A), Leu333-Ile315 (s5A-loops5A), Leu292-Met217 (s2C-s3C), Leu342-Val297 (s5A-s6A), Arg195-Tyr218 (s4C-s3C).

The disruption of the salt bridge between residues 289 and 362 caused distinct changes in the structure and stability of NS. The main structural parameters of E289A NS in comparison to WT NS were computed after a long simulation run and they are reported in the tabular scheme of Fig. 2. A notable difference was observed in the increased radius of gyration of E289A NS with respect to WT NS. This is likely due to the higher mobility of the RCL, which is not anchored by the salt bridge. The largest structural difference was in the number of hydrogen bonds (HBs) between main chain atoms (Table of Fig. 2). The picture in Fig. 2 shows HBs that were present with more than 50% occupancy in the WT and were lost in the E289A (blue ball and stick), and viceversa (red ball and stick). Interestingly, many of the main HBs that stabilised *β*-sheet A between strands s3A and s5A were lost in the mutant form of NS. As studied in our previous work^23^, the free-energy gain obtained in the conformational change from native to latent or cleaved NS is largely controlled by an increased number of HBs. Thus, we may argue that E289A NS may present a lower stability towards the non-native conformations that require the insertion of RCL as a strand in *β*-sheet A. More in general, HBs included in *α*-helical structure appear more persistent in WT NS, while HBs linking *β*-sheet strands have a relatively longer occupancy in E289A (as shown in Supporting Figure S1).

### Effects of the E289A mutation on protein dynamics

**Figure 3:**
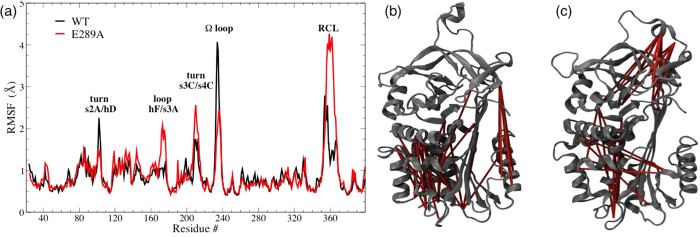
(**a**) Root Mean Square Fluctuations (RMSF) of each protein residue: WT NS (black line) and E289A NS (red line). The labels indicate the protein regions where WT and E289A NS display larger differences. (**b**,**c**) Cartoon representation of native NS and E289A NS, respectively, showing the most relevant dynamic correlations between non-consecutive residues (solid bars).

The analysis of root mean square fluctuations (RMSF) revealed other less expected effects of the E289A mutation. Figure 3a highlights the protein regions with the larger variation in RMSF of WT and E289A NS. Apart from the expected increase in RCL mobility, which is not anchored by the salt bridge in the E289A NS, we observed a decrease in the mobility of the Ω-loop in E289A NS. The Ω-loop is formed by a few residues between strands 1B and 2B and protrudes from the surface of the molecule and, in contrast to every other serpin, it regulates the inhibitory activity of NS against tPA, as proved before by deletion of this motif^9^. Figures 3b,c display as solid bars the pairs of residues exhibiting the strongest correlated motions (in terms of Linear Mutual Information^26^), and not belonging to the same secondary structure element. Operatively, this was implemented by taking into account correlated residues with a distance larger than 2 nm. As for the Ω-loop, the point mutation extends its effect towards distal regions of the protein. A striking effect is the loss of correlation between the main protein core and the F helix, which is a region important for polymerisation and activity^27^,^28^.

### *In vitro* mutagenesis of wild type neuroserpin

In order to address the predictions of the MD simulations, a recombinant variant of NS was produced in E. Coli by site directed mutagenesis changing the glutamic acid 289 into alanine, E289A NS. The conformation of the mutant protein and its functional and dysfunctional responses were tested *in vitro*.

### Kinetics of E289A neuroserpin polymerisation

**Figure 4:**
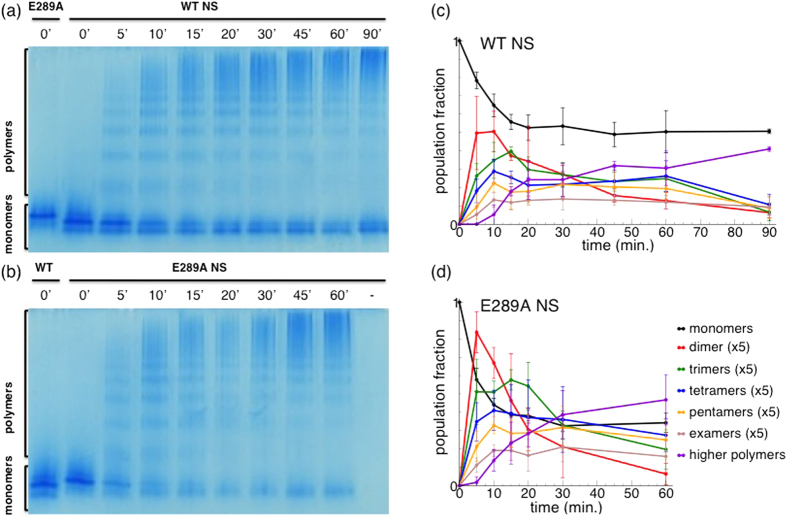
Polymerisation kinetics. (**a**,**b**) Non-denaturing PAGE of WT and E289A NS incubated at 52 °C for different time intervals (as reported in the figure). (**c**,**d**) Band density from non-denaturing PAGE as in panels (**a**,**b**). Points are normalised by the total density value of each lane. The points are averages over triplicate experiments. The values of dimer, trimer, tetramer and pentamer populations are scaled by a factor of 5 for easier visualisation.

The polymerisation of NS can be induced by moderate thermal stress. We incubated both WT and E289 NS at 52 °C for different time intervals and monitored the progress of polymerisation by non-denaturing poly-acrylamide gel electrophoresis (PAGE) (Fig. 4). The formation of small-size polymers produced the typical ladder-like pattern presented in Fig. 4a,b^29^. At the same time we observed the formation of latent NS, which run faster than native monomeric NS, due to its reduced hydrodynamic radius^23^. Both WT and E289A NS exhibited analogous polymerisation kinetics. A closer inspection by densitometry analysis (Fig. 4c,d) revealed that the polymerisation kinetics of E289A NS was slightly faster when compared to WT NS.

### Inhibitory activity of E289A neuroserpin

**Figure 5:**
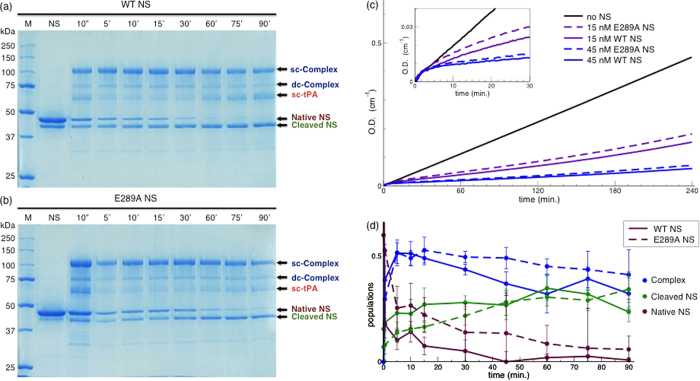
Inhibitory activity. (**a**,**b**) SDS-Page of 50 *μ*M WT NS and 50 *μ*M E289A NS incubated with 22 *μ*M tPA at 23 °C for different time intervals (as reported in the figure); the first lanes are the Molecular Weight markers (M), the second lanes are the native NS alone (NS). An arrow indicates the different species: double chain (dc) tPA/NS Complex, single chain (sc) tPA/NS Complex, single chain (sc) tPA, native NS, cleaved NS. The dc-Complex originates from the residual dc-tPA, and it looses the not bonded chain upon denaturation in reducing conditions. Analogously, the two chain of dc-tPA are separated upon denaturation with SDS and run with different velocity in the gel lanes (below 37 kDa). (**c**) Progress curves of hydrolysis of IPR-pNA (0.2 mM) by tPA (1 nM) in the presence of 0 NS (black line), 15 nM E289A NS (dotted violet line), 15 nM WT NS (solid violet line), 45 nM E289A NS (dotted blue line), 45 nM WT NS (solid blue line). (**d**) Band densities from SDS PAGE as in panel (a) for WT NS (solid lines) and panel (b) for E289A NS (dashed lines): NS/tPA Complex (blue lines), cleaved NS (green lines), native NS (brown lines). Points are normalised by the total density value of each lane. The points are averages over triplicate experiments.

The ability of E289A NS to form a complex with tPA was tested by mixing NS and tPA and incubating for different time intervals. The formation of the NS/tPA complex was monitored by SDS-PAGE (Fig. 5a,b). We clearly observed the rapid formation of the complex, and since the complex is known to be fragile at late stages^10^,^30^, we also observed the appearance of protein bands corresponding to cleaved NS and to non-complexed tPA, as well as the reduction in intensity of the complex. The densities of the gel bands in Fig. 5d did not display any significant difference in the ability to form the complex, while they showed a slower reduction of native NS for E289A NS with respect to the WT NS equivalent, in agreement with the chromogenic assay presented in Fig. 5c, which reports the progress of tPA inhibition by NS against a chromogenic substrate, IPR-pNA. The inhibitory activity of E289A NS was lower than that of WT NS. At later stages, our results suggest that tPA released after complex formation was still active, meaning that the protease is only partially translocated. Alternatively, this may suggest a disruption of the tPA-NS covalent bond and subsequent recovery of the integrity of the active site of tPA.

### Conformational stability of E289A neuroserpin

**Figure 6:**
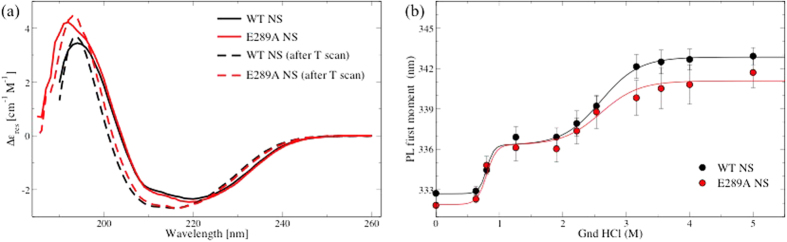
Thermal and chemical stability. (**a**) Far-UV CD spectra of WT NS (black lines) and E289A NS (red lines) at 25 °C before (solid lines) and after the temperature ramp from 25 to 90 °C at 1 °C/min. (**b**) First moment of emission spectra upon excitation at 275 nm as a function of Gnd HCl concentration: WT NS (black circles) and E289A NS (red circles). The points are averages over triplicate experiments. Solid lines are sigmoidal fit.

The correct folding of E289A NS was verified by far-UV circular dichroism (CD), a technique able to quantify the secondary structure content of protein folds. Figure 6a shows the CD spectra of both WT and E289A NS, which exhibited no differences, in agreement with the conformational stability in MD simulations. Also, the thermal stability of both NS variants, monitored by CD at 216 nm during a thermal ramp, displayed no relevant differences. Indeed, the main transition typically observed in a thermal ramp for NS is due to the formation of latent or polymer NS, rather than to thermal unfolding^18^,^21^. Figure 6a also shows the spectra for NS after a temperature ramp up to 90 °C, and therefore mainly related to polymeric NS, coherent with previously reported spectra of NS polymers^23^. Again, no significant changes were observed in the folding of both variants. The chemical stability of the native conformation was assessed by incubating NS with different amounts of a denaturant agent, guanidine hydrochloride (Gnd-HCl), and monitoring the intrinsic fluorescence of aromatic residues. In particular, the photoluminescence emission of tryptophans depends upon their exposure to a polar environment^31^; hence, the red-shift of the emission band (or more concisely of its first moment) can be used as a measure of protein unfolding^32^. Figure 6b shows that both E289A and WT NS presented a two-step unfolding transition at 0.8 M and 2.6 M Gnd-HCl concentration, as previously observed for WT NS^15^,^18^. In comparison to WT NS, E289A NS exhibited a decrease of cooperative behaviour to reach the final unfolding state.

## Discussion

MD simulations may play an important role in structural biology studies^33^. In protein science, they may complement the information obtained by X-ray crystallography or NMR. In our previous study^23^, we used MD to refine the structure of native and cleaved NS, and we uncovered some clues to the structure of the unsolved latent conformer. Here, we highlight a structural detail, not previously reported, that emerged from MD and was not resolved in crystallographic structures. A strong salt bridge links the Arg362 residue on the RCL to the Glu289 on strand s2C.

Analogously, another MD study of WT NS^22^ reported the formation of a strong hydrogen bond between Arg362 and Asp373 close to strand s1C. Such a difference with respect to our simulation may depend upon the choice of initial coordinates which were based upon the crystal structure of *Takehara et al. 2009*^9^. Indeed the two published structures of native WT NS^9^,^10^ exhibit larger differences in the RCL structure. Our MD work was based on the crystal structure of *Ricagno et al. 2009*^10^, where the RCL is completely resolved. We extended our MD work by performing the simulation of mutant Glu289 to Ala289 (E289A) and back to Glu289 (A289E), in order to validate the statistical significance of the presence of such a salt bridge and rule out the possible influence of initial coordinates. Our simulations and *in silico* mutations show that the Arg362 tends to approach residue Glu289, more than the region of Asp373 or the other RCL residues. The results of the simulations by Sarkar *et al.* and the present results both confirm that the RCL has a high probability of being anchored to the protein body by a strong hydrogen bond. In the present work we experimentally explored by point-site mutation the effects of the deletion of one anchor point by single aminoacid mutation of WT NS.

This salt bridge is located in a region that is critical for the inhibitory activity, which is close to the cleavage site. Therefore, we investigated the effect of the deletion of this salt bridge on protein structure and dynamics. From MD trajectories, we learned that such a point mutation may affect the dynamical behaviour of regions far from the mutated region. Interestingly, the mutation reduced the mobility (namely the RMSF) of the Ω-loop (Fig. 3), a region which is considered important for NS activity^9^. Also, it reduced the dynamical correlation between the main protein body and the F-helix, a region that is thought to be relevant for both activity and polymer formation^27^,^28^. A recombinant E289A variant of NS was produced in *E. coli* by site directed mutagenesis. We tested the conformational stability of the protein (Fig. 6a), its inhibitory activity (Fig. 5), its stability with respect to chemical denaturation (Fig. 6b), and its stability against thermal stress, that is, its propensity to polymer formation (Fig. 4). The removal of the salt bridge under analysis did not alter dramatically the native fold of NS. It altered the stability of NS in a subtle way. It slightly reduced the inhibitory efficiency and very slightly enhanced the polymerisation propensity, easing polymer formation and hindering the formation of a final complex with tPA. MD studies point to the Ω-loop and the F-helix as hot spots, responsible for setting a free-energy barrier between native NS and the unfolding state.

## Methods

### Mutagenesis, expression and purification of recombinant proteins

*E. coli* BL21 Rosetta 2 Competent Cells Novagen (Merck KGaA, Darmstadt, Germany) were transformed with the plasmid coding for human NS with a N-terminus 6-His tag in the pQE81L vector (Qiagen, Hilden, Germany) with ampicillin resistance. The plasmid for WT NS was kindly provided by D. Belorgey and D. Lomas, UCL, UK, and designed as in *Belorgey et al. 2002*^14^). The E289A mutation was introduced by polymerase chain reaction using the NS DNA template and specific mutagenic primers. The sequence of WT and mutant NS was confirmed by DNA sequencing of the entire NS gene.

The protocol for protein expression and purification was optimised from *Belorgey et al. 2002*^14^ and *Takehara et al. 2010*^20^. A seed culture was prepared by dropping a single colony from the transformed strain into 40 ml ampicillin-LB broth and shaking at 180 rpm for 16 hours at 37 °C. Cells were grown at 37 °C in 2xYT media containing 0.27 mM ampicillin by shaking at 180 rpm for about 4 hours until the O.D. at 600 nm reaches a value of 0.7 ± 0.2 *cm*^−1^, followed by isopropyl-*β*-D-thiogalactopyranoside (0.5 mM) induction, a further shaking at 150 rpm for 16 hr at 25 °C, and eventual cell harvesting by centrifugation at 3500 g for 10 minutes. The pelleted cells were washed twice with lysis buffer (50 mM Na_2_HPO_4_, 500 mM NaCl, pH 7.8), incubated on ice for 30 minutes upon addition of 1 tablet (for 5–10 grams of cells) of COMPLETE protease inhibitor cocktail (Hoffmann-La Roche, Basel, Switzerland) or/and 5 mM Aminoethyl-benzenesulfonyl fluoride hydrochloride, 1 mg/ml Lysozyme (Sigma-Aldrich, St. Louis MO, USA), 5 mM phenylmethylsulfonyl fluoride, and then disrupted by sonication at 4 °C (with long cooling intervals for each sonication cycle). NS was obtained from the soluble fraction by centrifugation at 12000 g for 15 minutes. All chemicals were purchased from Sigma-Aldrich, St. Louis MO, USA, unless differently specified.

The collected supernatant was loaded onto a 5 ml HiTrap Chelating HP Amersham column (GE Healthcare Europe GmbH, Freiburg, Germany) charged with 0.1 M NiSO_4_, and equilibrated with buffer A (20 mM Na_2_HPO_4_, 20 mM NaCl, pH 7.8), followed by buffer A containing 20 mM Imidazole. The protein was eluted with buffer A containing 300 mM Imidazole, diluted 4 times in 20 mM Tris-HCl, 20 mM NaCl, pH 7.4, loaded onto a UNO-Q6 column (Bio-Rad Laboratories, Segrate MI, Italy), and eluted with a linear NaCl gradient (20–1000 mM in 45 minutes). The collected fractions were assessed by 7.5% w/v non denaturing PAGE. Typically, the protein from a given elution peak was in monomeric form. This fraction was concentrated by Amicon 10 k Ultra-4 (Merck KGaA, Darmstadt, Germany) and buffer exchanged with the desired final buffer: 10 mM Na_2_HPO_4_, 100 mM NaCl, pH 7.4. Occasionally, a further purification was performed to isolate the monomeric form by gel filtration using a Hi Load 16/60 Superdex200 column (GE Healthcare Europe GmbH, Freiburg, Germany). NS concentration was measured by optical absorption using an extinction coefficient at 280 nm of 37000 *cm*^−1^ *M*^−1^ and a molecular weight of 46250 Da.

### Polymerisation assay

Solution of 10 *μ*M NS were incubated at 52 °C. At different time intervals, 10 *μ*l aliquots were put on ice and mixed with 1:1 loading buffer (250 mM Tris-HCl, 50% glycerol, 0.5% bromphenol blue, pH 6.8). Monomer and polymer NS were resolved on a 7.5% w/v non denaturant polyacrilamide gel electrophoresis (PAGE), and stained with PageBlue protein staining solution (Thermo Scientific, Waltham MA, USA). The density of the complex band was determined by densitometry scanning^29^.

### Activity Assays

The formation of NS/tPA complex was tested by incubating 50 *μ*M NS and 22 *μ*M two-chain and single chain recombinant tPA (either from American Diagnostica, Stamford CT, USA, or from Genentech, Inc. South San Francisco, CA, USA) at 23 °C in 50 mM Tris-HCl, 10 mM Na_2_HPO_4_, 200 mM NaCl, 0.1% Tween20, pH 7.4, for different time intervals. The reaction was stopped by adding 9:1 volumes of SDS loading buffer (10% SDS, 250 mM Tris-HCl, 50% glycerol, 0.5% bromphenol blue, 5% *β*-mercaptoethanol, pH 6.8) and boiled for 5 minutes. Proteins were resolved on a 10% w/v SDS PAGE and stained with PageBlue protein staining solution (Thermo Scientific, Waltham MA, USA). A protein standard broad range solution (Bio-Rad Laboratories, Segrate MI, Italy) was used as a reference on the first gel lane. The density of the complex band was determined by densitometry scanning and using the GelAnalyzer software (I. Lazar 2010 GelAnalyzer). For the chromogenic inhibitory assay NS at different concentrations (15 or 45 nM) was mixed with 200 *μ*M chromogenic substrate S-2288 (Cromogenix, Instrumentation Laboratory, Monza, Italy). The reaction was initiated by addition of 1 nM tPA and monitored by 410 nm absorption^10^,^15^,^20^,^22^.

### Thermal conformational stability

Circular dichroism (CD) spectra were recorded on 16 *μ*M NS solutions by a J-815 spectropolarimeter (Jasco, Tokyo, Japan) temperature controlled with a Peltier (using 0.01 cm quartz cuvettes, 3 nm bandwidth, 8 s response, 10 nm min^−1^ scan rate) and baseline-corrected by subtracting a buffer spectrum. The mean residue differential extinction coefficient Δ*ε*_*res*_ in *cm*^−1^ *M*^−1^ was calculated as in *Noto et al. 2015*^23^. Changes in the secondary structure with temperature were measured by monitoring the CD signal at 216 nm between 20 and 90 °C with a rate of 1 °C/min. The mid-point temperature *T*_1/2_ was calculated by fitting with a sigmoidal curve.

### Chemical conformational stability

Protein denaturation by guanidine hydrochloride (Gdn-HCl) was obtained after overnight incubation 4 °C of 3 *μ*M NS in 10 mM phosphate buffer, 100 mM NaCl, pH 7.40, in the presence of different amounts of the denaturant. At such a low temperature and low concentration, the formation of NS polymers upon denaturation is extremely delayed if present at all, as we checked by monitoring the mass and the hydrodynamic radius of NS incubated with denaturant with static and dynamic light scattering as previously described^19^. Fluorescence spectra were acquired at room temperature by a Jasco FP-6500 spectrofluorimeter (excitation wavelength 275 nm, response 2 s, 3 nm excitation bandwidth, 3 nm emission bandwidth, 100 nm min^−1^ scan rate), and baseline corrected by subtracting a buffer spectrum. The first moment *λ*_1_ of the normalised emission spectrum *L*(*λ*), defined as ∫*λL*(*λ*)d*λ*^32^, is related to the exposure of tryptophan residue and to the extent of protein unfolding^31^.

### Molecular Dynamics (MD)

MD calculations were performed on the IBM BCX/5120 cluster at CINECA. MD simulations of wild type native NS were described in *Noto et al. 2015*^23^. The initial molecular coordinates were based on the x-ray crystallographic coordinates by *Ricagno et al. 2009*^10^ (PDB: 3F5N) and on the analogy with *α*_1_-antitrypsin^25^ (PDB:1QLP) for the residues not completely solved in the NS crystal structure^23^. Native wild type NS was solvated by more than 12000 TIP3 water molecules^34^ and neutralised with the addition of 15 potassium ions in a rectangular simulation box of 9.3*x*7.3*x*7.2 *nm*^3^, after choosing the appropriate form for each histidine residue by a VMD tool^35^. MD trajectories were generated using the NAMD2 package^36^ and the Charm22 force field^34^, preceded by an adequate minimisation and equilibration steps, as described in *Noto et al. 2015*^23^. The simulation was performed in the NPT ensemble at 300 °C and atmospheric pressure, using periodic boundary conditions, a 2 fs time step along with the SHAKE algorithm to constrain the bond length of heavy atoms. Long-range electrostatic interactions were evaluated by the Particle Mesh Ewald method, while Van der Waals and Coulomb interactions were truncated using a switch function with a 1 nm cutoff. The simulation of E289A NS and A289E NS were performed by taking as initial coordinates the last run of WT NS and E289A NS, with boxes of 8.9*x*6.9*x*7.8 *nm*^3^ and 9.1*x*6.9*x*7.4 *nm*^3^, and with a neutralising number of potassium atoms of 14 and 15, respectively. Both simulations were run with the same conditions and protocols used for WT NS.

### Alchemical perturbation

After 45 ns of WT simulation, the glutamic acid residue 289 was changed into alanine, eliminating the salt bridge E289-R362. After 20 ns equilibration, a further 30 ns trajectory was generated and analysed. Eventually the alanine residue 289 was mutated back to glutamic acid and run for further 50 ns. The alchemical mutations for E289A and its reverse A289E were prepared using a VMD plugin and performed using NAMD2.

### Computational analytical tools

The Solvent Accessible Surface Area (SASA) was calculated on the protein heavy atoms by the NACCESS program [Hubbard, NACCESS 2.1.1 Univ. Manchester, UK, 1996], which estimates the accessible area by virtually rolling a probe of 1.4 Å around the molecular Van der Waals surface^37^. Hydrogen bonds analysis was carried out using a VMD plugin^35^. Hydrogen bonds were defined with a 3 Å cut-off distance between the donor (D) and the acceptor atom (A) and a D-H-A cut-off angle of 30°. The bond was classified as a salt bridge when the oxygen atom of acidic residues (Asp and Glu) and the hydrogen atoms of basic residues (Arg, Lys and the protonated His) are within a 3 Å cut-off distance. The Wordom package^38^ was used to build the covariance matrix of coordinate fluctuations: *C*_*ij*_(*δ***r**_*i*_, *δ***r**_*j*_) where *δ***r**_*i*_ = **r**_*i*_ − 〈*δ***r**_*i*_〉, **r**_*i*_(*t*) is the Cartesian coordinate of atom *i* and the angular brackets 〈〉 represent the time average over the trajectory. Before computing the covariance matrix elements *C*_*ij*_ the protein coordinates are fit to a reference structure to remove the global translational and rotational motion. A correlated motion between pair of residues is evaluated by using the linear mutual information method of *Lange and Grubmuller 2006*^26^, which provides linear correlations among coordinates by calculating the opportune coefficient from the covariance matrix.

## Additional Information

**How to cite this article**: Noto, R. *et al.* The stability and activity of human neuroserpin is modulated by a salt bridge that stabilises the reactive centre loop. *Sci. Rep.* **5**, 13666; doi: 10.1038/srep13666 (2015).

## Supplementary Material

Supplementary Information
