# Supplementary material for: The stability and activity of human neuroserpin are modulated by a salt bridge that stabilises the reactive centre loop
Source: Sci Rep. 2015 Sep 2;5:13666. doi: 10.1038/srep13666 (PMC4556959; doi:10.1038/srep13666)
Supplement: Supplementary Information [file srep13666-s1.pdf]

SUPPORTING INFORMATION

The stability and activity of human neuroserpin is modulated by a salt bridge that stabilises the reactive centre loop

Rosina Noto[1], Loredana Randazzo[1], Samuele Raccosta[1], Sonia Caccia[2], Claudia Moriconi[3], Elena Miranda[3], Vincenzo Martorana[1], and Mauro Manno[1,*]

[1]National Research Council of Italy, Institute of Biophysics, Palermo, Italy

[2]Department of Medical Biotechnologies and Translational Medicine, University of Milan, Milan, Italy

[3]Department of Biology and Biotechnologies "Charles Darwin" and Pasteur Institute - Cenci Bolognetti Foundation, Sapienza University of Rome, Rome, Italy

*mauro.manno@cnr.it

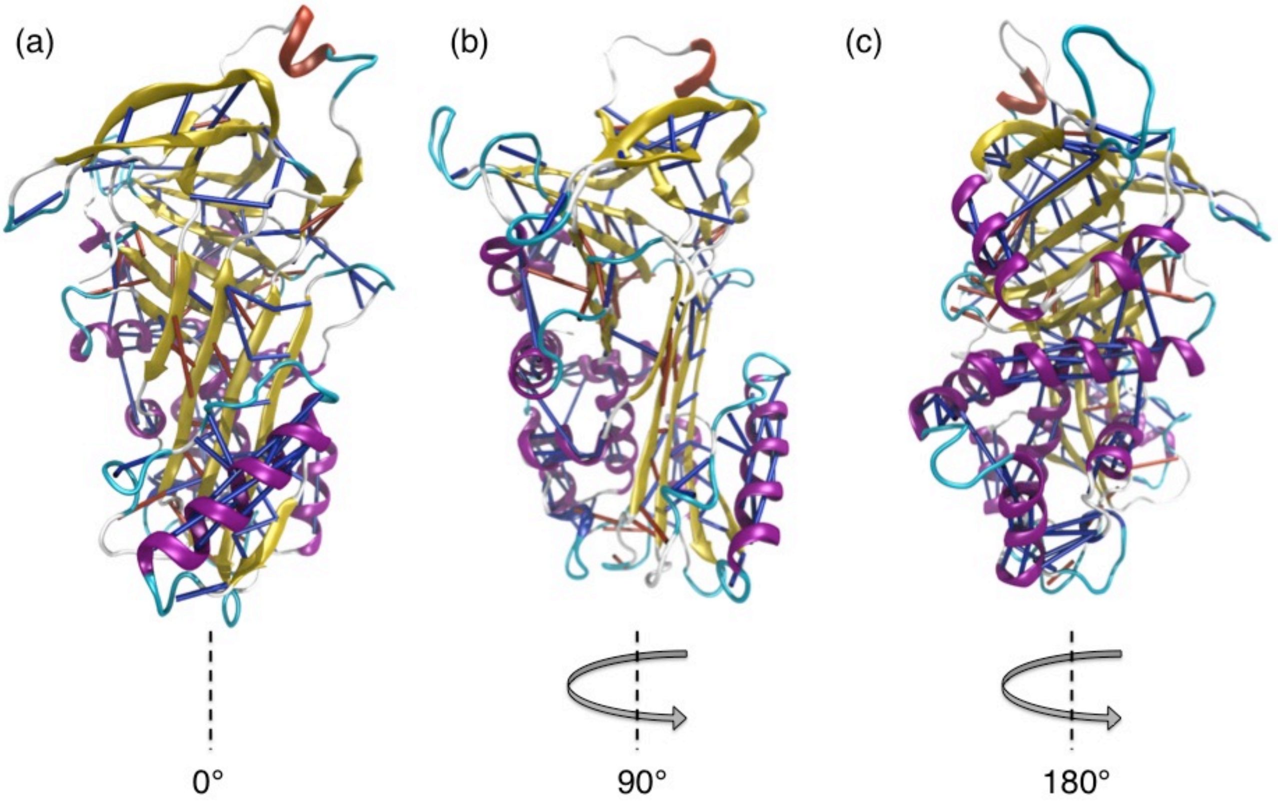

Supporting Figure S1. Cartoon representation of WT NS showing a selection of hydrogen bonds (HBs) as blue and red sticks. (a) Frontal view with the F-helix on the foreground; (b) 90° rotation with respect to (a); (c) 180° rotation with respect to (a). Blue sticks: HBs that in WT NS have an occupancy time higher than 50% with respect to the occupancy time in E289A NS. Red sticks: HBs that in E289A NS have an occupancy time higher than 50% with respect to the occupancy time in WT NS.
